# The Interference Pattern of Plasmonic and Photonic Modes Manipulated by Slit Width

**DOI:** 10.3390/nano10040730

**Published:** 2020-04-11

**Authors:** Xing Li, Jing Tang, Xuelian Zhang, Ruirui Zhang, Xiangyu Zeng, Zijun Zhan, Chunxiang Liu, Chuanfu Cheng

**Affiliations:** Shandong Provincial Engineering and Technical Center of Light Manipulations & Shandong Provincial Key Laboratory of Optics and Photonic Device, College of Physics and Electronics, Shandong Normal University, Jinan 250014, China; tjjsdnu@126.com (J.T.); a1173176125@163.com (X.Z.); zhangruirui0268@163.com (R.Z.); zengxiangyu0611@163.com (X.Z.); zhanzijun1990@live.cn (Z.Z.); liuchunxiang@sdnu.edu.cn (C.L.)

**Keywords:** surface plasmon, interference pattern, polarization, slit width

## Abstract

We demonstrate that the interference pattern of the plasmonic and photonic modes can be controlled by changing the slit width of a square slit structure. Based on the analyses of the plasmonic and photonic modes of slits with different widths, we theoretically derived the expressions of wavefield generated by a square slit. A far-field scattered imaging system is utilized to collect the intensity distribution experimentally. Various interference patterns, including stripes, square-like lattice array, and diamond-like lattice array, have been observed by adjusting the slit widths. In addition, the results were validated by performing finite-difference time-domain simulations, which are consistent with the theoretical and experimental results.

## 1. Introduction

Propagating along the two-dimensional (2D) metal/dielectric interface, surface plasmon polaritons (SPPs) with the subwavelength, field confinement, and field enhancement features have been regarded as a powerful platform to realize nanocircuit for on-chip communication and computation [1,2,3]. Nanodevices with various functionalities, ranging from focusing [4,5,6,7], vortex generation [8,9,10,11], and hologram generation [12,13] to light bending [14,15,16,17], unidirectional propagation [18,19,20,21], and logic operation [22,23],are demonstrated.

The manipulation of the plasmonic field is an important subject in up-to-date plasmonic optics. To control the plasmonic field, the shapes, widths, and orientations of slits are the elements to control the launched wavelets [18,24,25,26,27], and the interferences of the wavelets form the wave field patterns [8,9,10,11,12,28,29,30]. The interferences can be described intuitively by the Huygens–Fresnel principle for subwavelength metal slits [31,32], and the principle has facilitated the manipulations of the plasmonic field. Moreover, the polarization of incident light has a great influence on the generation of SPPs. Specifically, only the incidence of transverse magnetic (TM) polarization, which is perpendicular to the slit structure, can excite plasmonic modes, while transverse electric (TE) polarizations excite waves of photonic modes [33,34,35]. Slits under arbitrary linearly polarized illuminations may excite wavelets of the two modes. The two modes have been used in the design of nanoslit lenses with polarization-selective [33]. Generally, the component quantities of excitation efficiencies, the initial phases, and the equivalent wavevectors of the two modes depend highly on the slit widths [28,33,34], and the differences in the quantities would apparently influence the wave field. This dependence and the interference of the wavelets are the foundation for the formation of wavefield patterns.

A method for obtaining the solution of the photonic mode and the plasmonic mode waves produced by slits with fixed width and fixed polarization of the incident waves was studied in Ref. [36]. However, the dependence of the two mode waves on slit width and its influence on the interference pattern need to be further investigated. As a basic subject for nanostructure design and wavefield manipulations, modulating the interference pattern with slit widths is worth studying. In this paper, the interference patterns generated by square slits with different silt widths are theoretically and experimentally studied. The amplitude and phase features of a single rectangular slit are firstly analyzed with finite-difference time-domain (FDTD) simulations. Then, the square slits are fabricated with the focused ion beam (FIB) method and characterized with a scattered imaging system. For vertically polarized incident light, the interference patterns evolve from horizontal stripes to dot arrays and are observed with the increase of the slit width. A square-like dot array or diamond-like dot array can be selectively obtained for the different width square slit illuminated by the 45° linearly polarized light. Simulations of the interference patterns are consistent with the theoretical and experimental results. The generation of 2D surface optical lattices can find potential applications for defect detection in semiconductor devices, improving the alignment resolution in nanofabrication techniques [37] and two-dimensional particle capture and manipulation [29].

## 2. Results and Discussions

**Figure 1:**
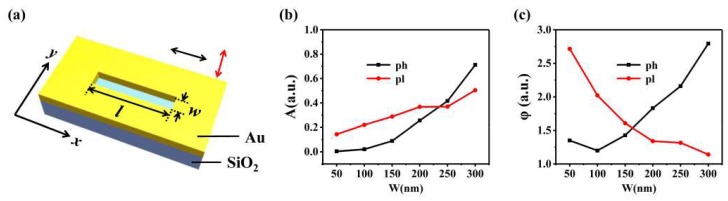
Schematic diagram of a metal rectangle slit (**a**–**c**) are the amplitude and phase of the plasmonic (red) and photonic (black) modes generated by slits with different widths.

Figure 1a schematically shows a rectangular slit etched on a 200 nm gold film, and the substrate is silicon. The plasmonic mode and photonic mode can be correspondingly excited when they are illuminated by TM (red arrow) and TE (black arrow) polarized light [33,34,35]. The features of these two modes, including amplitude and phase, strongly depend on the slit width [28,33,34]. With commercial software FDTD solutions, the plasmonic mode and photonic mode generated by slits with different widths are analyzed. The length of the slit is a fixed value *l* = 8 μm and the width *w* of the slit changes from 50 to 300 nm with an interval of 50 nm. The wavelength of the incident light is 632.8 nm. The simulated amplitude and the simulated phase of these two modes are and shown in Figure 1b,c, respectively. It can be seen from Figure 1b that the amplitude of both the plasmonic mode and photonic mode increases linearly with the slit width. When the slit width is less than 100 nm, the amplitude of the photonic mode is almost zero because the incident wave cannot transmit through the slit. The amplitude value of the photonic mode wave is equal to, and then exceeds, that of the plasmonic mode when the width approaches 230 nm. In Figure 1c, we see that the initial phase of the photonic mode increases with the increase in the slit width, while the initial phase of the plasmonic mode decreases with the increase in the slit width. One point should be noted that the polarization direction of the excited plasmonic and photonic mode waves is the same as that of corresponding incident light [36].

**Figure 2:**
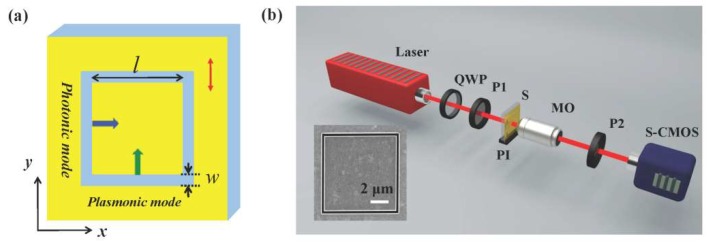
(**a**) schematically shows the square slit. With vertically incident light, photonic mode and plasmonic mode waves are excited by the two vertical slits and two horizontal slits, respectively. (**b**) is the scattered imaging system utilized to experimentally characterize the fabricated square slit (inset).

Based on the above analyses of a single rectangle slit, we study the optical field generated by a square slit, which is schematically shown in Figure 2a. The slit has a fixed length of *l* = 8 μm, and the width of the slit increases from *w* = 100 nm to *w* = 200 nm with an interval of 50 nm. For the vertically polarized incident wave (the red arrow), the plasmonic modes (the blue arrow) are excited by upper and lower horizontal arms, and the photonic modes (the green arrow) are excited by left and right vertical arms. For an arbitrary field point (*x*,*y*) in the central area of the square slit, the optical field generated by the two horizontal slits and two vertical slits can be correspondingly expressed as [24]:

(1) $$\begin{array}{l} E_{pl}(x,y)=A_{pl}\;\exp(i\Phi_{pl}-ik_{pl}r),\\ E_{ph}(x,y)=A_{ph}\;\exp(i\Phi_{ph}-ik_{ph}r). \end{array}$$

The subscript *pl* and *ph* represent the plasmonic mode and the photonic mode, respectively, and *k*, *r*, *A,* and Φ denote the wave vector, the distance between slit and field point (*x*,*y*), the initial amplitude, and the phase, respectively. The initial amplitude and phase of the two modes can be obtained from the simulation results in Figure 1b,c. Considering that the polarization of the excited plasmonic and photonic mode waves is the same as that of the incident light, the polarization of both $E_{pl}(x,y)$ and $E_{ph}(x,y)$ are along the *y* direction. Then, the optical field in the center area is the interference of the two plasmonic waves and the two photonic waves, which can be written as:

(2) $$E(x,y)=E_{pl}^{upper}(x,y)+E_{pl}^{lower}(x,y)+E_{ph}^{left}(x,y)+E_{ph}^{right}(x,y).$$

Substituting Equation (1) into Equation (2), we obtain the following:

(3) $$E(x,y)=2A_{ph}\cos(k_{ph}y+\Phi_{ph})+2A_{pl}\cos(k_{pl}x+\Phi_{pl}).$$

**Figure 3:**
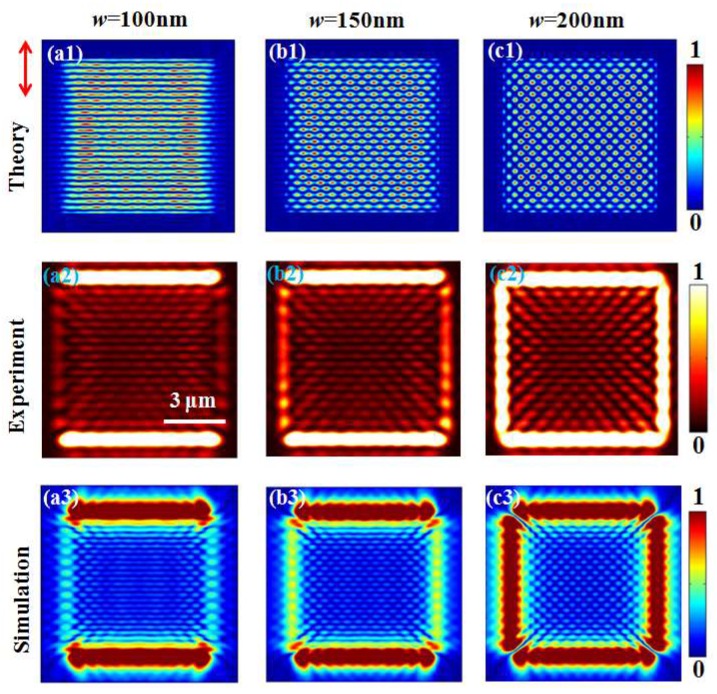
For vertically polarized incident light (indicated by the red arrow), (**a1**)–(**c1**) describe the normalized theoretical intensity distributions of $|E(x,y){|}^{2}$ generated by the square slits with different widths. The optical patterns evolve from horizontal stripes to diamond-like dot array with the increase in slit width. (a2)–(c2) and (a3)–(c3) are the corresponding experimental and finite-difference time-domain (FDTD) results. The slit widths of square-slit samples are 100 nm, 150 nm and 200 nm, respectively.

Considering that the values $A_{ph}$, $A_{pl}$, $\Phi_{ph}$, $\Phi_{pl}$ in Equation (3) vary with the slit width, as analyzed in the above context, the optical field of E(x,y) may have different expressions for different slit widths. Therefore, different types of interference patterns can be realized by selecting the slit width of the square slits. With Equation (3), the intensity distributions $|E(x,y){|}^{2}$ generated by the square slit with different widths are theoretically acquired, which are given in Figure 3a. It is interesting to see that the patterns evolve from horizontal stripes to 2D dot array as the width increases from 100 to 200 nm. For the 100 nm slit in Figure 3a1, the amplitude of the plasmonic mode is about 10 times stronger than the photonic mode, as shown in Figure 1b. Thus, the optical patterns mainly result from the interference of two plasmonic modes generated by the horizontal slits. The propagation direction of SPPs generated by the upper and lower slits are opposite and therefore horizontal standing SPP waves with a period of about half of the SPP wavelength (300 nm) are formed. When the slit width is 200 nm in Figure 3c1, the amplitude of the photonic mode excited by the vertical slits increases and is about equal to the amplitude of the plasmonic mode excited by the horizontal slits. In this case, a uniform dot array pattern resulting from the interference between a horizontal standing wave and a vertical standing wave is observed. For the 150-nm square slit, the interference pattern takes on a mixture of horizontal stripes and dot array, as shown in Figure 3b1.

A scattered imaging experimental setup [36] in Figure 2b is built up to characterize the optical pattern generated by the square slit. The light source is a linearly polarized He-Ne laser with a wavelength of 632.8 nm. A quarter wave plate (QWP) and a polarizer (P1) are utilized to adjust the polarization of the light incident on the sample (S). The sample was placed on a three-dimensional positioning system (PI, P-611.3S) for precision position adjustment. The light of plasmonic mode and photonic mode is collected with a microscopic objective (MO, Carl Zeiss AG, Jena, Germany, N.A. = 0.9/100×), another polarizer (P2), and an S-CMOS (Zyla-5.5, 16-bit). The square slit samples are fabricated using a focused ion beam (FIB) system, and a scanning electron microscope (SEM) image of a fabricated square slit with a width of 200 nm is given in the inset of Figure 2b. The experimental results in Figure 3b show that the interference patterns evolve from horizontal stripes to 2D dot array, which is similar to the above theoretical results. The differences in the borders of the maps, corresponding to the slits between theoretical and experimental intensity distributions, are caused by the approximation of slit wave source in theoretical calculation. The slits with different widths in the experiments are approximated as linear light sources with “zero” slit width. The diffraction effects caused by slits are not taken into account, which makes the borders of the maps look different. However, the amplitude and phase of photonic and plasmonic waves generated by the approximated slit are the same as those generated by actual slits in Figure 1b,c. Thus, the calculated interference patterns in the center area should reproduce the experimental results. 

To further verify the theoretical results and experimental results, we performed simulations of the square slit structures with FDTD solutions. In the simulation, the square slit structures have the same structural parameters as those used in the theoretical calculations. The vertically polarized plane wave is normally incident from the bottom of the silicon along the *z* axis. The thickness of the gold film is set as 200 nm, which can prevent the incident light from transmitting through the metal film. In simulations, the field monitor is set to be at the height of 450 nm above the sample surface, which is the same position where the amplitude and phase of these two modes generated by the single slit in Figure 1b,c are obtained. At such a height around one wavelength, both the plasmonic mode and photonic mode are neither too strong nor too weak, which makes the simulation data more robust. We can see that the simulated intensity patterns in Figure 3a3–c3 are consistent with the theoretical results and experimental results. It is noticed that experimental intensity patterns are obviously larger than the FDTD-simulated results. The differences in size can be explained by the imaging characteristics of our experimental detection system. When the interference wavefield is formed on the film surface, the slightly rough surface formed in the film growth will scatter a small fraction of wavefield into the air, and it is collected by the scattered imaging detection. According to the scattering theory under Kirchhoff’s approximation [38], the scattered wave from a microfacet will introduce an additional decrease $-k_{sz}\zeta_{rms}$ of wave vector with the root-mean-squares lope $\zeta_{rms}$ of the slightly rough film surface [39]. The scattered the wavevectors component of the plasmonic mode and photonic mode are written as $k_{s,pl}=k_{pl}-k_{sz}\zeta_{rms}$ and $k_{s,ph}=k_{ph}-k_{sz}\zeta_{rms}$, respectively. The period of observed interference pattern will be greater than that of the metal surface due to the decrease $-k_{sz}\zeta_{rms}$ in $k_{pl}$ and $k_{ph}$. 

The 2D interference patterns of the square slits can be further controlled by introducing more plasmonic modes and photonic modes with appropriate incident polarization, for instance, a diagonal linear polarization. For the incident plane wave with a diagonal linear polarization, it can be decomposed into a horizontal and a vertical component:

(4) $$E_{0}{=E}_{0x}+E_{0y}=E_{0}(e_{x}+e_{y}).$$

When the *x*-polarized component $E_{0x}$ excites the plasmonic waves from vertical slits and photonic waves from horizontal slits, the y-polarized component $E_{0y}$ can excite the plasmonic waves from horizontal slits and photonic waves from vertical slits. Thus, the vector form of wavefields E(x,y) produced by the square slit can be represented as:

(5) $$\begin{array}{cc} E(x,y)=E_{x}(x,y)e_{x}+E_{y}(x,y)e_{y} & =\left[E_{ph}^{upper}(x,y)+E_{ph}^{lower}(x,y)+E_{pl}^{left}(x,y)+E_{pl}^{right}(x,y)\right]e_{x}\\ & +\left[E_{pl}^{upper}(x,y)+E_{pl}^{lower}(x,y)+E_{ph}^{left}(x,y)+E_{ph}^{right}(x,y)\right]e_{y}. \end{array}$$

From the above equation, it can be seen that the plasmonic mode and the photonic mode waves can be excited simultaneously at any of the four slits. The *x*-polarized field component $E_{x}(x,y)$ is the interference of the photonic modes generated by the upper and lower slits and the plasmonic modes generated by the left and right slits, while the y-polarized field component $E_{y}(x,y)$ is the interference of the plasmonic modes excited by the upper and lower slits and the photonic modes excited by the left and right slits. After substituting Equation (1) into Equation (5), we can obtain:

(6) $$\begin{array}{cc} E(x,y)=E_{x}(x,y)e_{x}+E_{y}(x,y)e_{y} & =\left[2A_{ph}\cos(k_{ph}y+\Phi_{ph})+2A_{pl}\cos(k_{pl}x+\Phi_{pl})\right]e_{x}\\ & +\left[2A_{pl}\cos(k_{pl}y+\Phi_{pl})+2A_{ph}\cos(k_{ph}x+\Phi_{ph})\right]e_{y}, \end{array}$$

where the parameters $A_{ph},\;A_{pl},\;\Phi_{ph},\;\Phi_{pl}$ significantly influence the physical meaning of the formula and the formation of the 2D interference patterns. It is known that the values $A_{ph},\;A_{pl},\;\Phi_{ph},\;\Phi_{pl}$ in Equation (6) can be changed by adjusting the slit width of the square slit structures. Then, by selecting an appropriate slit width of the square slit structures, the expected 2D interference patterns may be obtained.

**Figure 4:**
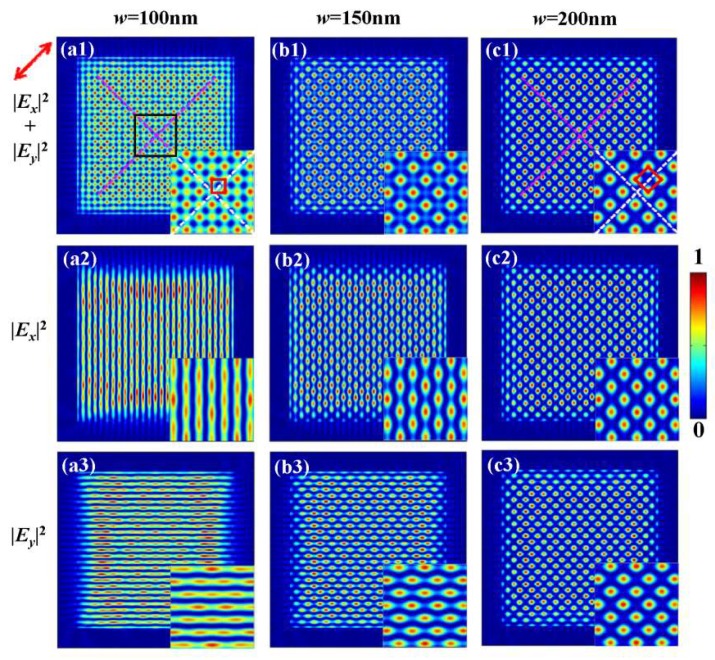
(**a**–**c**) Normalized theoretical intensity patterns for the square slits with *w* = 100, 150, 200 nm, respectively. (**a1**–**c1**) are the total intensities $|E_{x}(x,y){|}^{2}+|E_{y}(x,y){|}^{2}$. (**a2**–**c2**) are the intensities of the *x*-polarized patterns $|E_{x}(x,y){|}^{2}$. (**a3**–**c3**) are the intensities of the *y*-polarized patterns $|E_{y}(x,y){|}^{2}$. The insets in each image are the enlarged map of the area within the black box. The red arrow represents the polarization direction of the incident light.

**Figure 5:**
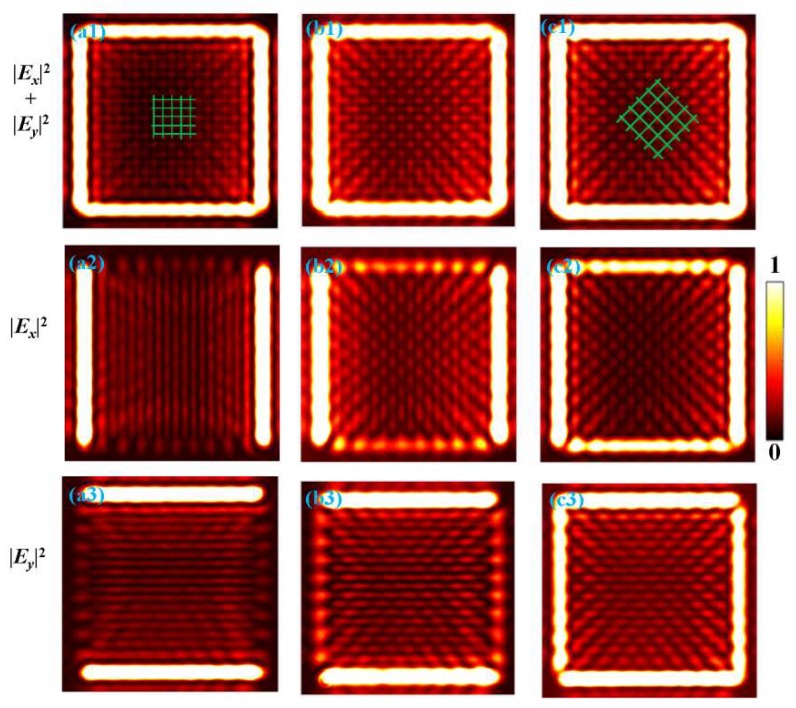
(**a**–**c**) Experimentally obtained patterns of the square slit samples with *w* = 100, 150, 200 nm, respectively. (**a1**–**c1**) are the patterns of the total intensities $|E_{x}(x,y){|}^{2}+|E_{y}(x,y){|}^{2}$, and (**a2**–**c2**) are the intensities of the x-polarized patterns $|E_{x}(x,y){|}^{2}$. (**a3**–**c3**) are the intensities of y-polarized patterns $|E_{y}(x,y){|}^{2}$. The square-like pattern and diamond-like pattern are indicated by the green lines.

Using the analytical solution Equation (6), the calculated intensity distributions are presented in Figure 4. Figure 4a1–c1 shows the map of the total intensity $|E_{x}(x,y){|}^{2}+|E_{y}(x,y){|}^{2}$. Figure 4a2–c2 and a3–c3 present the component intensity maps of $|E_{x}(x,y){|}^{2}$ and $|E_{y}(x,y){|}^{2}$, respectively. The insets in images are the corresponding magnified patterns within the black box, and the red squares indicate their character. From the patterns in Figure 4a1–c1, we can see that 2D intensity patterns of optical lattices are different. To clarify the characteristics of these optical lattices, we define the space between the adjacent maximum points of the intensity as the periodicity *d* of the lattice pattern. For the slit width of 100 nm in Figure 4a1, the SPP interference field is a square-like lattice array oriented parallel to the coordinate system and the spacing is d≈300 nm. For the slit width of 200 nm in Figure 4c1, a distinct diamond-like lattice array can be observed and the spacing is d≈425 nm along the diagonals. For the slit width of 150 nm in Figure 5b1, the intensity pattern is the superposition of the two types of patterns described above. The dependence of the two orthogonal components $|E_{x}(x,y){|}^{2}$ and $|E_{y}(x,y){|}^{2}$ on the width are also studied, as shown in Figure 4a2–c2 and a3–c3. With the increase in the slit width, the $|E_{x}(x,y){|}^{2}$ component patterns evolve from vertical stripes to the diamond-like dot array, and the $|E_{y}(x,y){|}^{2}$ also evolves from horizontal stripes to the diamond-like dot array, which is similar to the evolution of intensity patterns generated under *y*-polarized illumination. 

This evolution can be quantitatively explained by two cases for Equation (6). First, when the photonic mode is much weaker than the plasmonic mode wave, i.e., when $A_{ph}:A_{pl}\to 0$, Equation (6) can be simplified as:

(7) $$E(x,y)=\left[2A_{pl}\cos(k_{pl}x+\Phi_{pl})\right]e_{x}+\left[2A_{pl}\cos(k_{pl}y+\Phi_{pl})\right]e_{y}.$$

The field component $E_{x}(x,y)$ becomes the interference of plasmonic waves excited by two vertical slits and the component $E_{y}(x,y)$ is also the interference of plasmonic waves by two horizontal slits, as shown in Figure 4a2,a3. The interference field E(x,y) in this situation can be regarded as the superposition of two orthogonal plasmonic standing waves. Thus, the total intensity distributions $|E_{x}(x,y){|}^{2}+|E_{y}(x,y){|}^{2}$ can bewritten as $4A_{pl}^{2}\left\{{\left[\cos(k_{pl}x+\Phi_{pl})\right]}^{2}+{\left[\cos(k_{pl}y+\Phi_{pl})\right]}^{2}\right\}$, indicating that the field pattern excited by the four slits will be the square spot array, as represented in Figure 4a1. Second, when the initial amplitudes of these two mode waves are relatively close, i.e., when $A_{ph}:A_{pl}\to 1$, Equation (6) can be converted to:

(8) $$E(x,y)=2A_{pl}\left\{\begin{array}{l} \left[\cos(k_{ph}y+\Phi_{ph})+\cos(k_{pl}x+\Phi_{pl})\right]e_{x}\\ +\left[\cos(k_{pl}y+\Phi_{pl})+\cos(k_{ph}x+\Phi_{ph})\right]e_{y} \end{array}\right\}$$

In this case, the field component $E_{x}(x,y)$ is the interference between a horizontal plasmonic standing wave and a vertical photonic standing wave, and the field component $E_{y}(x,y)$ is the interference between a vertical plasmonic standing wave and a horizontal photonic standing wave. The slight difference between the wave vectors $k_{pl}$ and $k_{ph}$ only affects the position of the interference fields and not the shape of the field pattern. Moreover, it is known that the initial phase difference $\Phi_{ph}-\Phi_{pl}$ between two-mode waves determines the field distribution pattern, and the field component pattern is a diamond-like dot pattern when $\Phi_{ph}-\Phi_{pl}=0$ [30]. The field $|E_{x}(x,y){|}^{2}$ and $|E_{y}(x,y){|}^{2}$ in Figure 4c2,c3 shows as a diamond-like dot pattern with $\Phi_{ph}-\Phi_{pl}=\pi /2$. When the condition $\Phi_{ph}-\Phi_{pl}=0$ is approximately satisfied, as the superposition of $|E_{x}(x,y){|}^{2}$ and $|E_{y}(x,y){|}^{2}$, the total intensity distributions $I_{tot}$ forms a uniform diamond-like array pattern, as shown in Figure 4c1. When $A_{ph}:A_{pl}$ is an arbitrary value between 0 and 1, the resulting interference field from the four slits will be transitional patterns between the square-like spot array and the diamond-like spot array patterns, corresponding to Figure 4b1.

Illuminated by the diagonally polarized light, the experimental patterns for the square slits with *w*=100, 150, 200nm are shown in Figure 5a–c. By rotating polarizer P2, the patterns of components $|E_{x}(x,y){|}^{2}$ and $|E_{y}(x,y){|}^{2}$ are obtained, which are shown in Figure 5b1–b3 and c1–c3, respectively. Figure 5a1–a3 are obtained without P2 and represents the total intensity $|E_{x}(x,y){|}^{2}+E_{y}(x,y){|}^{2}$. We see that all the calculated and experimental patterns of total and polarized intensities are of high consistency.

**Figure 6:**
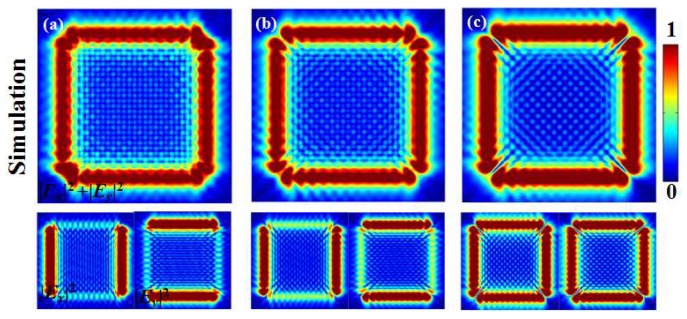
(**a**–**c**) are the corresponding simulation results with finite-difference time-domain (FDTD).

Finally, the corresponding FDTD simulation results are shown in Figure 6a–c. The upper large images show the patterns of the total intensities $|E_{x}(x,y){|}^{2}+E_{y}(x,y){|}^{2}$, and the lower-left and lower-right patterns in each figure are patterns of intensities $|E_{x}(x,y){|}^{2}$ of x-polarization and $|E_{y}(x,y){|}^{2}$ of y-polarization, respectively. The simulated intensity patterns are consistent with the theoretical results and experimental results, demonstrating the feasibility of the method and its ability to manipulate lattice patterns.

## 3. Conclusions

In conclusion, with the scattering imaging system, we have experimentally demonstrated the manipulation of interference patterns by varying the slit width. The square slit structures of different slit widths are characterized. Based on the excitation of the plasmonic and the photonic modes, we theoretically analyze the physical mechanism of generating the interference fringes and the interference spots array. We have shown that the experimental patterns are in a good agreement with both the analytical and simulated results. Potential applications of the interference patterns in this manuscript include defect detection in semiconductor devices, improving the alignment resolution in nanofabrication techniques [37] and particle trapping and manipulation [29].
